# Quadriceps muscle volume has no effect on patellofemoral cartilage lesions in patients with end-stage knee osteoarthritis

**DOI:** 10.1186/s43019-022-00134-6

**Published:** 2022-02-19

**Authors:** Jung-Ro Yoon, Hong Joon Joo, Seung Hoon Lee

**Affiliations:** Department of Orthopedic Surgery, Veterans Health Service Medical Center, 53 Jinhwangdo-ro 61-gil, Gangdong-gu, Seoul, 05368 Republic of Korea

**Keywords:** Patellofemoral cartilage lesion, Quadriceps, Alignment, Hip–knee–ankle, *Q*-angle

## Abstract

**Purpose:**

The quadriceps muscle has a positive effect on anterior knee pain. However, its effect on the patellofemoral (PF) cartilage in patients with end-stage knee osteoarthritis is unknown. The present study aimed to evaluate whether the quadriceps muscle area had a positive effect on the PF cartilage and whether this muscle had a positive effect on the clinical scores.

**Materials and methods:**

Patients with confirmed cartilage status and clinical scores who underwent total knee arthroplasty (TKA) were included. The PF cartilage status was evaluated during TKA. The thickness and the area of the quadriceps muscle were measured using a knee computed tomography scan obtained before the surgery. The *Q*-angle, hip–knee–ankle angle, alignment, and Insall–Salvati ratio were measured by radiography.

**Results:**

Altogether, 204 patients were included in the study. Logistic regression was performed including factors associated with PF cartilage lesions. The regression model was found to be statistically significant (Hosmer–Lemeshow test, *χ*^2^ = 0.493). A smaller hip–knee–ankle (HKA) angle was associated with a higher incidence of PF cartilage lesions (*p* = 0.033) and only the alignment had an effect on the PF cartilage lesions. PF cartilage lesions did not correlate with the clinical scores. A thicker medial portion of the quadriceps muscle was associated with a significantly higher Knee Society Knee Score (KSKS) (*p* = 0.028).

**Conclusions:**

Quadriceps muscle thickness and area, *Q*-angle, and patellar height were not associated with PF cartilage lesions, while a smaller HKA angle was associated with PF cartilage lesions. The presence of PF cartilage lesions did not affect the clinical symptoms. However, a thicker medial portion of the quadriceps muscle was associated with a higher KSKS.

## Introduction

Anterior knee pain is one of the main types of knee pain in elderly individuals, and patellofemoral (PF) cartilage lesions are the representative causes [1, 2]. Even in patients with end-stage knee osteoarthritis (OA) undergoing total knee arthroplasty (TKA), anterior knee pain due to PF cartilage lesions results in significant morbidity. Conservative treatments are performed for most PF cartilage lesions [3]. Among the conservative treatment approaches, quadriceps exercises can be easily performed by the patients. The quadriceps muscle affects the PF joint through the magnitude and direction of its force. Several studies have addressed the relationship between the quadriceps muscle power and the PF joint. Most of the studies reported that lower quadriceps muscle power was associated with greater damage to the PF cartilage or higher PF joint pressure [4, 5]. However, some studies have reported contrasting observations [6, 7].

Several factors such as the *Q*-angle, alignment, and patellar height can also affect PF cartilage lesions. An increased *Q*-angle could lead to a high lateral PF contact pressure [8]. Another study suggested that a high *Q*-angle was unlikely to be associated with changes in the thickness of the knee articular cartilage [9]. The patellar height did not correlate with PF articular cartilage congruence [10]. However, a further study reported that abnormal patellar height was significantly correlated with chondral lesions [11].

To date, there is no clear consensus on the effects of the quadriceps muscle, *Q*-angle, and patellar height on the PF joint. Therefore, the effect of quadriceps exercises on PF cartilage lesions remains unclear. In addition, recent studies mainly targeted the younger age groups, and PF cartilage status was measured using radiologic images. The present study aimed to evaluate whether the quadriceps muscle area had a positive effect on the PF cartilage and whether this muscle had a positive effect on the clinical scores. The hypotheses of this study were as follows: (1) the quadriceps muscle, *Q*-angle, alignment, and patellar height affect the PF cartilage lesions and (2) differences are observed in symptoms according to the presence of PF cartilage lesions, and the symptoms are affected by the quadriceps muscle.

## Materials and methods

### Patients

All patients who underwent primary TKA between March 2019 and August 2020 were retrospectively reviewed. Among these, patients with medial knee OA on simple anteroposterior knee radiographs were included for comparison with cartilage conditions other than those involving the PF cartilage. All patients who underwent TKA between March 2019 and August 2020 were included. The exclusion criteria were as follows: (1) patients with inflammatory and traumatic arthritis, (2) patients with previous knee joint fractures, (3) patients who underwent ligament or cartilage surgery, (4) patients who underwent surgery that could affect the lower extremity alignment except for the knee, and (5) patients with valgus alignment of the lower extremities.

Clinical scores were measured in all patients before the surgery and preoperative knee computed tomography (CT) was performed. PF cartilage lesions were evaluated during the surgery. Approval from the Institutional Review Board was prospectively obtained before the analysis (approval number: BOHUN 2021-01-028).

### Cartilage evaluation

The cartilage status was evaluated during TKA. For each patella and trochlea, areas with cartilage damage were classified according to the International Cartilage Repair Society scoring system [12]. Grades 1 and 2 were assumed to indicate no PF cartilage lesions, while a higher degree of damage was assumed to indicate advanced PF cartilage lesions.

### Radiographic evaluation

**Fig. 1:**
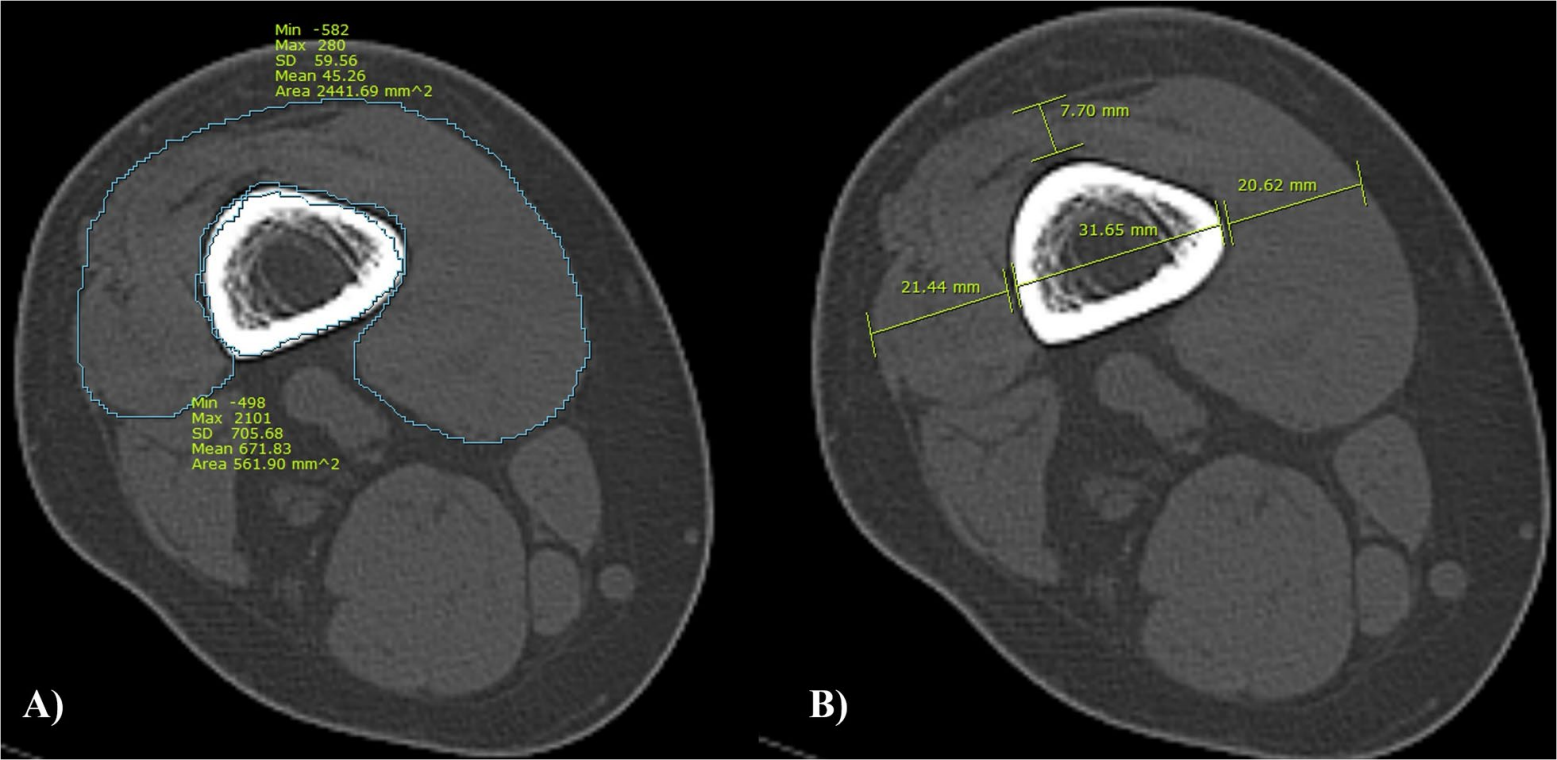
Quadriceps muscle thickness and area at 3 cm proximal to the patellar upper pole. **A** Measurement of the quadriceps and femoral area. **B** The widest axis of the femur was assumed to represent the width. The medial and lateral thickness of the quadriceps muscle was measured on the extension line of the width

**Fig. 2:**
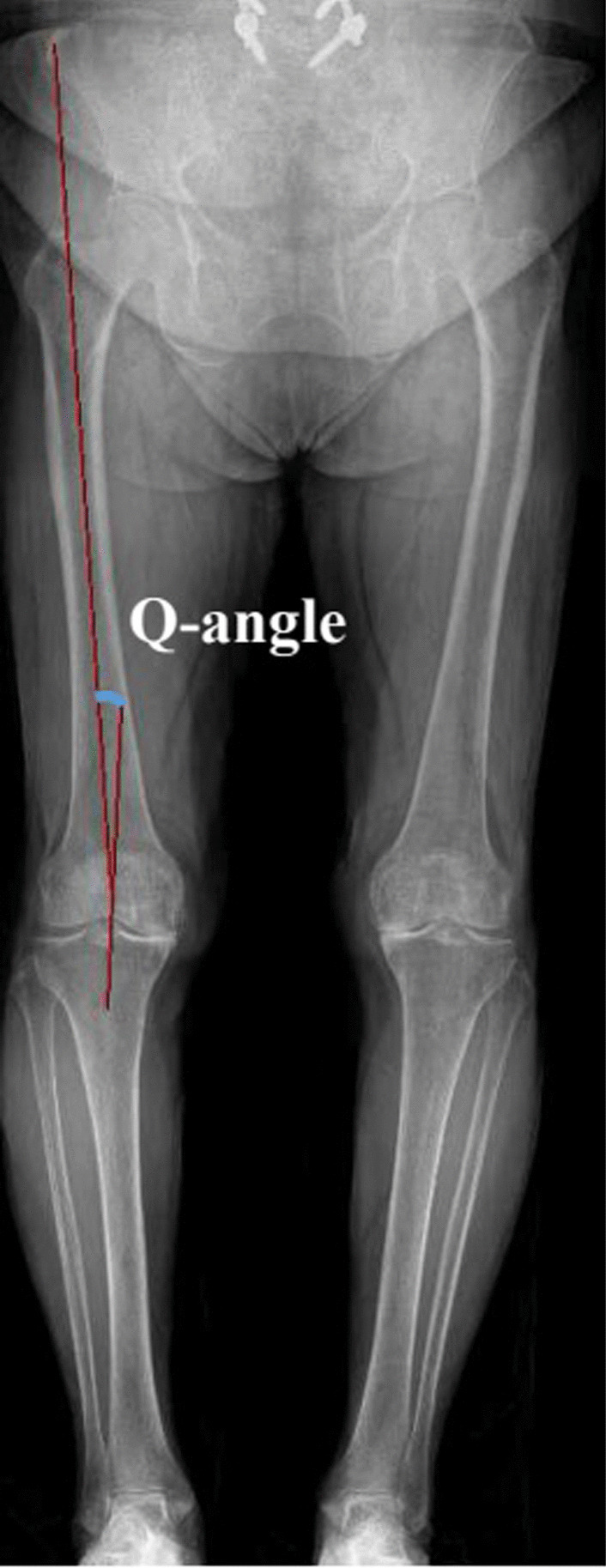
Measurement of the *Q*-angle. The angle between the line connecting the center of the patella and the tibial tuberosity, and the line connecting the center of the patella and the anterior superior iliac spine was defined as the *Q*-angle

**Fig. 3:**
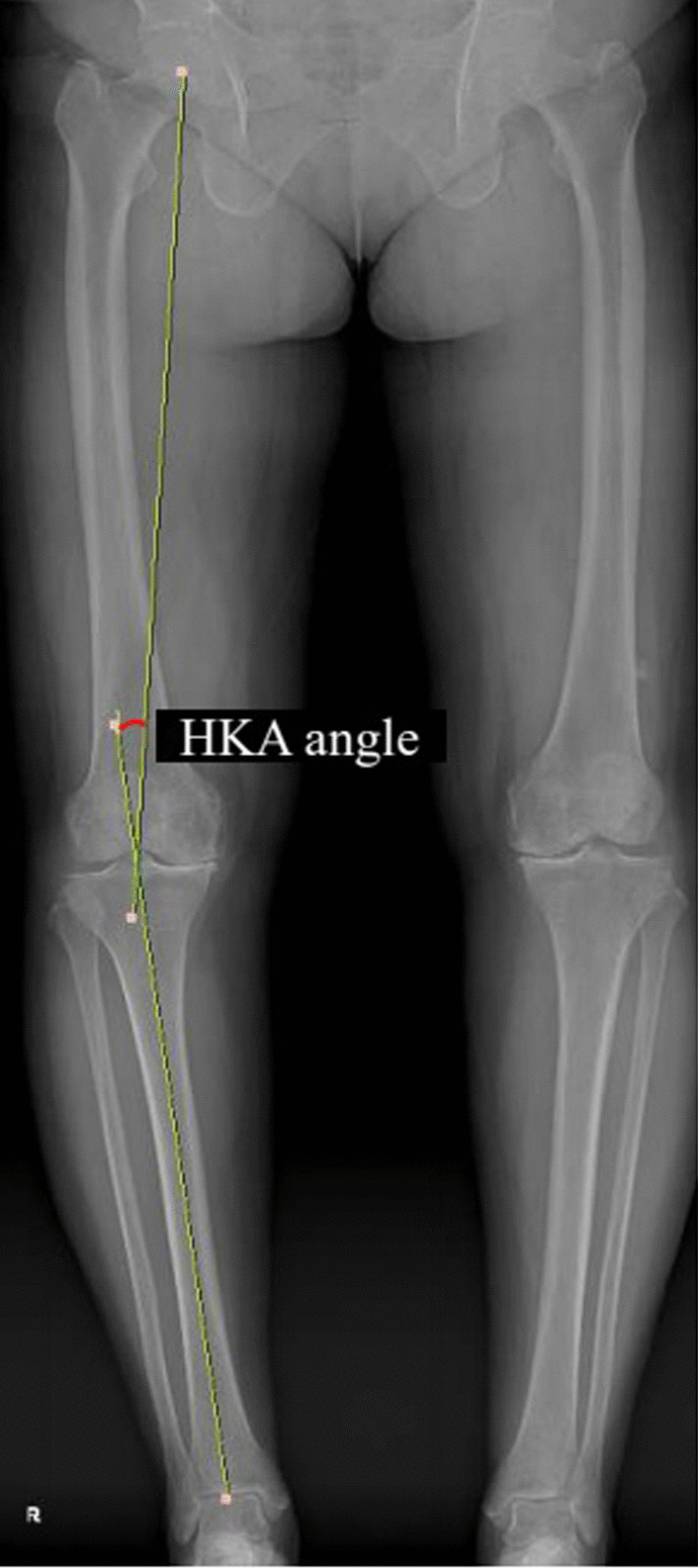
Measurement of the hip–knee–ankle angle. The angle between the line connecting the center of the femoral head and the intercondylar center of the femur, and the line connecting the center of the tibial spine and the center of the talar dome was defined as the hip–knee–ankle angle

**Fig. 4:**
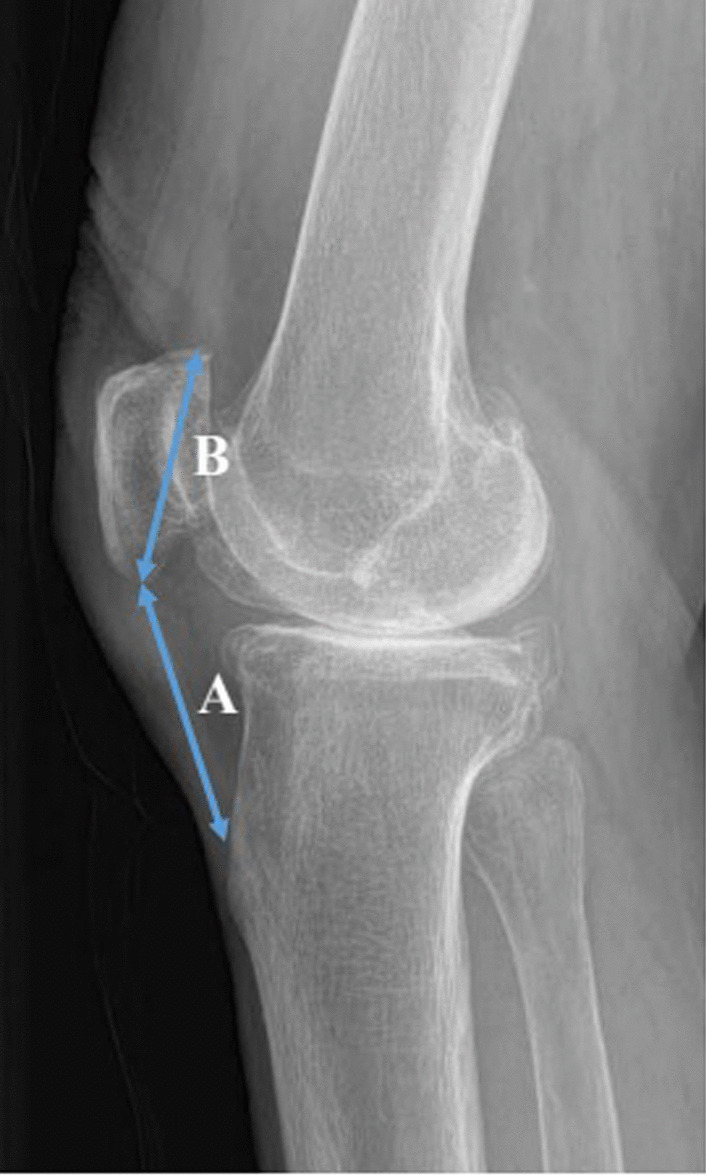
The Insall–Salvati ratio. The ratio was represented as *B*/*A*

The quadriceps muscle was evaluated using a knee CT scan obtained before the surgery [13, 14]. The thickness and area of the quadriceps muscle were measured at 3 cm from the upper pole of the patella. Femoral width and area were also measured at the same level to account for individual physical characteristics. The medial, anterior, and lateral thickness of the quadriceps muscle was also measured to determine the relative quadriceps strength [15]. While measuring the thickness of the quadriceps muscle, the widest axis of the femur was assumed to indicate the width. The medial and lateral thickness of the quadriceps muscle was measured on the extension line of the width (Fig. 1). The anterior thickness was measured at the midpoint of quadriceps. To measure the direction of the *Q*-muscle vector, the angle between the patellar tendon and the line connecting the patella and the anterior superior iliac spine (the *Q*-angle) was measured (Fig. 2). The hip–knee–ankle (HKA) angle was also measured (Fig. 3). The Insall–Salvati ratio was measured using lateral knee radiography to calculate the patellar height (Fig. 4). All radiographic parameters were measured twice at 1-month intervals by one of the surgeons (L.S.H.) and the mean values were used for the analysis. The intrarater reliability of the measurements was assessed using the intraclass correlation coefficient (ICC). The aforementioned measurements were performed using the INFINITT picture archiving and communication system (INFINITT Healthcare, Seoul, South Korea).

### Clinical score evaluation

Numeric pain rating scale score, Western Ontario and McMaster Universities Osteoarthritis (WOMAC) score, Knee Society Functional Score, and Knee Society Knee Score (KSKS) were measured by an orthopedic resident to confirm the patients’ clinical symptoms before the surgery [16, 17].

### Statistical analysis

All statistical analyses were performed using SPSS Statistics (version 22.0; IBM Corp., Armonk, NY, USA). Logistic regression analysis was performed including the Insall–Salvati ratio, alignment, *Q*-angle, muscle area, and thickness to identify the factors affecting PF cartilage lesions. The difference in the clinical scores according to the presence or absence of PF cartilage lesions was analyzed using an independent *t*-test. The relationship between the muscle area and thickness with the clinical scores was analyzed using simple correlation analysis. The statistical significance was set at *p* < 0.05.

## Results

A total of 262 patients met the inclusion criteria. Among these, 58 patients (1 with inflammatory arthritis, 2 with traumatic arthritis, 5 with a previous knee fracture, 8 with a previous knee ligament or cartilage surgery, 30 with other surgeries that could affect the alignment, and 12 with valgus alignment) were excluded, and 204 patients were finally included in this study (Table 1). A total of 100 patients had no PF cartilage lesions, while 104 patients had advanced PF cartilage lesions. The ICC values of all measurements were 0.8 or higher (Table 1).

**Table 1:** Patient demographics and radiologic evaluation

	No PF cartilage lesion (*N* = 100)		Advanced PF cartilage lesion (*N* = 104)		*p*-Value	ICC
	Average	SD	Average	SD		
Age (years)	72.9	5.4	74.3	4.9	0.061	
Height (cm)	156.8	8.4	158.1	7.8	0.289	
Weight (kg)	64.8	10.9	67.2	10.3	0.100	
HKA angle	9.0	4.7	7.4	4.8	0.020	0.92
Insall–Salvati ratio	1.1	0.1	1.1	0.2	0.515	0.84
*Q*-angle	−0.9	4.8	−1.5	4.3	0.304	0.82
*Q*-muscle area (mm^2^)	2519.2	650.4	2568.2	638.9	0.595	0.91
Femur area (mm^2^)	801.1	138.6	893.3	574.1	0.127	0.92
*Q*-muscle thickness: medial (mm)	20.9	5.5	21.6	6.7	0.447	0.93
*Q*-muscle thickness: anterior(mm)	11.4	4.1	11.2	3.3	0.760	0.88
*Q*-muscle thickness: lateral (mm)	17.7	4.9	19.0	10.6	0.290	0.91
Femur width (mm)	35.5	4.0	36.2	3.4	0.191	0.89

*HKA* hip–knee–ankle, *Q-muscle* quadriceps muscle, *PF* patellofemoral, *SD* standard deviation, *OA* osteoarthritis, *ICC* intraclass correlation coefficient

### Relationship between patellofemoral cartilage lesions and the quadriceps muscle

Logistic regression was performed, including factors believed to affect the PF cartilage lesions. The regression model was found to be statistically significant (Hosmer–Lemeshow test, *χ*^2^ = 0.493). However, the explanatory power was low (Nagelkerke’s *R*^2^ = 5.6). There was a tendency for the absence of PF cartilage lesions with an increase in the HKA angle (*p* = 0.033). Except for the alignment, none of the factors had any effect on PF cartilage lesions (Table 2).

**Table 2:** Logistic regression analysis for factors affecting patellofemoral arthritis

	B	S.E	OR	95% CI	*p*-Value
Insall–Salvati ratio	0.413	0.911	1.512	0.254 ~ 9.013	0.650
HKA angle	−0.082	0.039	0.921	0.854~0.994	0.033
*Q*-angle	0.021	0.040	1.022	0.945~1.105	0.589
*Q*-muscle area: Femur bone area	−0.043	0.237	0.958	0.602~1.525	0.857
Medial *Q*-muscle thickness: femur width	−0.435	1.249	0.647	0.056~7.483	0.727
Anterior *Q*-muscle thickness: femur width	−1.265	1.308	0.282	0.022~3.661	0.333
Lateral *Q*-muscle thickness: femur width	0.569	0.929	1.767	0.286~10.907	0.540

−2LL = 263.210, NagelKerke *R*^2^ = 0.056, Hosmer–Lemeshow test: *χ*^2^ = 3.201 (*p* = 0.921), *Q-muscle* quadriceps muscle, *HKA* hip–knee–ankle

### Relationship between clinical symptoms and the quadriceps muscle

PF cartilage lesions did not correlate with the clinical scores (Table 3). Analysis of the association between clinical scores and the area and thickness of the quadriceps muscle revealed that the KSKS tended to be significantly higher in the medial portion of the quadriceps (*p* = 0.028). A thicker medial portion of the quadriceps muscles tended to be associated with lower WOMAC scores, but the association was not statistically significant (*p* = 0.070) (Table 4).

**Table 3:** Comparison of clinical scores according to patellofemoral arthritis

	No. PF cartilage lesion (*N* = 100)		Advanced PF cartilage lesion (*N* = 104)		*p*-Value
	Average	S.E	Average	S.E	
Pain NRS	5.09 [4–6]	0.32	5.05 [5–6]	0.22	0.279
WOMAC	59.46 [44–78]	10.1	60 [50–75]	9.35	0.692
KSFS	54.16 [19–60]	9.41	54.88 [32–60]	6.66	0.525
KSKS	39.56 [30–70]	7.75	38.95 [28–60]	7.39	0.567

*PF* patellofemoral, *OA* osteoarthritis, *NRS* numeral rating scale, *WOMAC* Western Ontario and McMaster Universities Osteoarthritis index, *KSFS* Knee Society Functional Score, *KSKS* Knee Society Knee Score

**Table 4:** Correlation analysis of clinical scores, and quadriceps muscle area and thickness

	*Q*-muscle area: femur bone area	Medial *Q*-muscle thickness: femur width	Anterior *Q*-muscle thickness: femur width	Lateral *Q*-muscle thickness: femur width
Pain NRS	−0.003 ± 0.031 (*p* = 0.919)	0.028 ± 0.112 (*p* = 0.805)	0.076 ± 0.099 (*p* = 0.442)	−0.036 ± 0.073 (*p* = 0.622)
WOMAC	−0.419 ± 1.071 (*p* = 0.696)	−7.041 ± 3.866 (*p* = 0.070)	−1.712 ± 3.427 (*p* = 0.618)	−2.027 ± 2.529 (*p* = 0.424)
KSFS	0.326 ± 0.874 (*p* = 0.710)	−5.238 ± 3.155 (*p* = 0.099)	−0.413 ± 2.797 (*p* = 0.883)	3.155 ± 2.064 (*p* = 0.128)
KSKS	−0.772 ± 0.833 (*p* = 0.356)	6.680 ± 3.009 (*p* = 0.028)	2.192 ± 2.667 (*p* = 0.412)	−1.275 ± 1.968 (*p* = 0.518)

*Q-muscle* quadriceps muscle, *NRS* numeral rating scale, *WOMAC* Western Ontario and McMaster Universities Osteoarthritis index, *KSFS* Knee Society Function Score, *KSKS* Knee Society Knee Score

## Discussion

The principal findings of this study were as follows: (1) The thickness and area of the quadriceps muscle, *Q*-angle, and patellar height were not associated with PF cartilage lesions, while a smaller HKA angle alignment was associated with PF cartilage lesions. (2) The presence of PF cartilage lesions did not affect the clinical symptoms in patients with medial knee OA. However, a thicker medial portion of the quadriceps muscle tended to be associated with a higher KSKS.

This study aimed to improve the symptoms of OA by analyzing the effect of the quadriceps muscle, which is one of the modifiable factors causing PF cartilage lesions. Among factors such as the direction, point of action, and magnitude of the quadriceps muscle force, the magnitude of the muscle force can be modified in elderly patients with medial knee OA, but not the direction or the point of action. However, in the present study, quadriceps muscle area and thickness did not affect the PF cartilage lesions in patients with medial knee OA. Among the analyzed factors, alignment was the only factor that affected the PF cartilage lesions. However, since surgery is the only way to correct the alignment in elderly patients, this finding is not helpful for the patients. Quadriceps muscle thickness and area, which are modifiable factors, were not associated with PF cartilage lesions.

In the present study, a smaller HKA angle was associated with a lower incidence of PF cartilage lesions. Although the effect of alignment on PF cartilage lesions is not known, it has been reported that varus alignment affects PF arthritis of the lateral facet in patients with end-stage knee OA [18]. The authors presumed that the *Q*-muscle force vector changes according to the alignment, resulting in PF cartilage lesions. However, the exact mechanism of alignment-dependent PF cartilage lesions cannot be identified from the study results alone.

In the present study, the presence of PF cartilage lesions did not affect the clinical symptoms. There has been a debate about the effectiveness of patellar resurfacing during TKA [19, 20]. However, the contribution of PF cartilage lesions to pain in patients with medial knee OA is important [21]. In the present study, there was no difference in the clinical scores according to the presence of PF cartilage lesions in patients with medial knee OA. Research on the quadriceps muscle change and anterior knee pain after TKA without patellar resurfacing could determine the effect of the quadriceps muscle on anterior knee pain more precisely.

In cases of medial knee OA, high tibial osteotomy is sometimes performed to correct the alignment [22, 23]. Lateral release and cartilage regeneration are sometimes performed to treat PF cartilage lesions [24]. However, in many cases, conservative treatment is performed, especially for patients with end-stage knee OA. Exercise is a type of conservative treatment that elderly patients can perform on their own to relieve symptoms. Particularly, quadriceps exercises are widely taught to patients with knee pain [3]. However, there is a controversy regarding the idea that quadriceps muscle exercises improve knee pain [3, 5, 25]. The results of the present study might provide a theoretical basis for quadriceps muscle exercises in patients with knee OA.

Unlike previous studies, the present study involved evaluation of the actual PF cartilage rather than radiological evaluation, which might be an advantage over other studies. However, this study has some limitations. First, the authors used the general knee score and not the anterior knee pain score. Not using a score indicating anterior knee pain might be a disadvantage in terms of evaluating PF cartilage lesions. Second, the present study was conducted using the area and thickness of the quadriceps muscle measured using CT, but not the power of the quadriceps muscle. The muscle measurements on CT may be inaccurate and the measured value may not represent the actual quadriceps muscle power. In addition, the thickness of the medial and lateral muscles may not accurately represent the thickness of the vastus medialis and vastus lateralis muscles, respectively. Third, the effect of patellar and trochlear shapes on PF cartilage lesions was not considered in the present study. Fourth, the retrospective design of the study might have led to selection bias. However, we attempted to reduce bias as much as possible through the exclusion criteria. Fifth, the muscle area and width were measured at 3 cm above the upper pole of the patella. This position itself cannot be a clear reference point for muscle measurement.

## Conclusions

Quadriceps muscle thickness and area, *Q*-angle, and patellar height were not associated with PF cartilage lesions, while a smaller HKA angle associated with PF cartilage lesions. The presence of PF cartilage lesions did not affect the clinical symptoms. However, a thicker medial portion of the quadriceps muscle was associated with a higher KSKS.

## Data Availability

The datasets analyzed during the current study are available from the corresponding author on reasonable request.

## Abbreviations

CT: Computed tomography
HKA: Hip–knee–ankle
ICC: Intraclass correlation coefficient
KSKS: Knee Society Knee Score
OA: Osteoarthritis
PF: Patellofemoral
TKA: Total knee arthroplasty
WOMAC: Western Ontario and McMaster Universities Osteoarthritis
